# Sleep patterns in midlife and Alzheimer's disease biomarkers

**DOI:** 10.1002/alz70860_100535

**Published:** 2025-12-23

**Authors:** Clémence Cavaillès, Mercedes Carnethon, Kristen L Knutson, Stephen Justin Thomas, Kristine Yaffe

**Affiliations:** ^1^ University of California, San Francisco, San Francisco, CA, USA; ^2^ Northwestern University Feinberg School of Medicine, Chicago, IL, USA; ^3^ University of Alabama at Birmingham, Birmingham, AL, USA

## Abstract

**Background:**

Growing evidence suggests an association between sleep and Alzheimer's disease (AD) among older adults. However, the relationships between sleep patterns and AD blood biomarkers earlier in life remain underexplored. We aimed to investigate how sleep in early midlife relates to three AD biomarkers in late midlife.

**Method:**

We analyzed 1,332 participants from the Coronary Artery Risk Development in Young Adults (CARDIA) study. At the study baseline (2000‐2001), participants answered binary questions about their typical sleep patterns, including general sleep quality, sleep duration, daytime sleepiness, snoring, and insomnia symptoms (e.g., trouble falling asleep, waking up at night, and early awakening). On average 15 years later (2015‐2016), plasma β‐amyloid (Aβ) 42/40 and phosphorylated tau (*p*‐tau)217 were assayed using the Fujirebio Lumipulse G1200 analyzer, while neurofilament light chain (NfL) were analyzed using the Quanterix Simoa HD‐X assay. Lower Aβ42/40 and higher *p*‐tau217 and NfL levels are indicative of a higher AD risk. Square root and log transformations were applied to NfL and *p*‐tau217 respectively to improve the normality of the distributions. We used multivariable linear regressions to examine the standardized associations between sleep patterns and AD biomarker levels.

**Result:**

At baseline, the participants’ mean age was 40.3 (±3.5) years, 57% were women, and 43% were Black. Around 15% of participants reported a short sleep duration (<6 hours), 15% poor sleep quality, 54% at least one insomnia symptom, 26% daytime sleepiness, and 75% snoring. After adjusting for age, sex, race/ethnicity, education, and study center, poor sleep quality was linked to higher level of NfL (β=0.16, 95% 95% confidence interval (CI)=0.01,0.30). Daytime sleepiness was associated with lower Aβ 42/40 (β=‐0.13, 95% CI=‐0.25,‐0.01) and higher NfL (β=0.17, 95% CI=0.05,0.29) levels. Additionally, snoring was associated with lower level of Aβ 42/40 (β=‐0.15, 95% CI=‐0.28,‐0.02).

**Conclusion:**

These findings indicate that sleep disturbances in early midlife is associated with unfavorable levels of key plasma AD biomarkers, indicative of higher risk of AD, 15 years later. This study supports prior evidence suggesting sleep disturbances as potential modifiable risk factors for AD.

